# Complete genome sequence of the deep South China Sea-derived *Streptomyces niveus* SCSIO 3406, the producer of cytotoxic and antibacterial marfuraquinocins

**DOI:** 10.1371/journal.pone.0248404

**Published:** 2021-03-23

**Authors:** Qinghua Zhu, Weige Cheng, Yongxiang Song, Qing He, Jianhua Ju, Qinglian Li

**Affiliations:** 1 College of Life Science, Dezhou University, Dezhou, China; 2 CAS Key Laboratory of Tropical Marine Bio-Resources and Ecology, Guangdong Key Laboratory of Marine Materia Medica, RNAM Center for Marine Microbiology, South China Sea Institute of Oceanology, Chinese Academy of Sciences, Guangzhou, China; Universidad Nacional Autonoma de Mexico Facultad de Quimica, MEXICO

## Abstract

*Streptomyces niveus* SCSIO 3406 was isolated from a sediment sample collected from South China Sea at a depth of 3536 m. Four new sesquiterpenoid naphthoquinones, marfuraquinocins A-D, and two new geranylated phenazines, i. e. phenaziterpenes A and B, were isolated from the fermentation broth of the strain. Here, we present its genome sequence, which contains 7,990,492 bp with a G+C content of 70.46% and harbors 7088 protein-encoding genes. The genome sequence analysis revealed the presence of a 28,787 bp gene cluster encoding for 24 open reading frames including 1,3,6,8-tetrahydroxynaphthalene synthase and monooxygenase, seven phenazine biosynthesis proteins, two prenyltransferases and a squalene-hopene cyclase. These genes are known to be necessary for the biosynthesis of both marfuraquinocins and phenaziterpenes. Outside the gene cluster (and scattered around the genome), there are seven genes belonging to the methylerythritol phosphate pathway for the biosynthesis of the essential primary metabolite, isopentenyl diphosphate, as well as six geranyl diphosphate/farnesyl diphosphate synthase genes. The strain *S*. *niveus* SCSIO 3406 showed type I PKS, type III PKS and nonribosomal peptide synthetase cluster. The sequence will provide the genetic basis for better understanding of biosynthesis mechanism of the above mentioned six compounds and for the construction of improved strain for the industrial production of antimicrobial agents.

## Introduction

**Fig 1:**
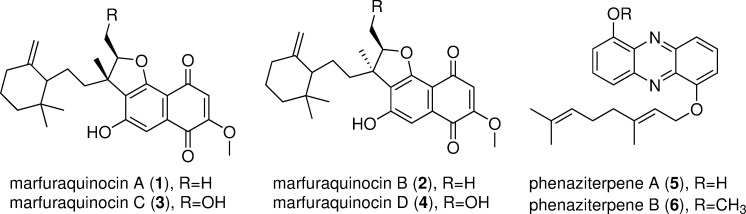
The chemical structure of compounds marfuraquinocins A-D (1–4) and phenaziterpenes A-B (5–6).

Deep-sea *Streptomyces* are widely recognized as an emerging source of novel and bioactive secondary metabolites [1]. They have been phylogenetically classified in 13 groups (MAR1-MAR13) [2]. The MAR4 group is a rich source of polyketide-terpenoid secondary metabolites, such as marinone, azamerone, and napyradiomycins [3–5]. As a member of the MAR4 group, *Streptomyces* sp. CNQ-509 could produce two polyketide-terpenoids (naphterpin and debromomarinone), five new farnesyl-α-nitropyrroles nitropyrrolins A–E and O-prenylated phenazines marinophenazine A-B [6]. Here, *S*. *niveus* SCSIO 3406 *w*as isolated from a South China Sea sediment sample collected at a depth of 3536 m. Four new sesquiterpenoid naphthoquinones, marfuraquinocins A-D (**1**–**4**) (Fig 1), which exhibited antibacterial activities against *Staphylococcus aureus* ATCC 29213 or methicillin-resistant *Staphylococcus epidermidis* (MRSE) were previously isolated from the strain SCSIO 3406 [7]. Additionally, two new geranylated phenazines, phenaziterpenes A and B (**5**–**6**) (Fig 1), were also isolated from this strain in spite of low production [7]. In order to gain insights about the genetic basis of the above six compounds and about the discovery of further new natural products, the genome of *S*. *niveus* SCSIO 3406 was sequenced.

## Materials and methods

*S*. *niveus* SCSIO 3406 was cultivated in trypticase soy broth and grown for 2 days at 28°C with 200-rpm aeration. High-molecular-weight DNA was prepared using standard genomic DNA isolation method [8].

Genomic DNA was then used to generate two libraries, one is paired-end (PE) library with insert sizes of 300~500 bp and the other is a SMRTbell^TM^ template library with insert sizes of about 8~10 kb. The complete genome of *S*. *niveus* SCSIO 3406 was subsequently sequenced using a combination of PacBio RSII sequencing (Pacific Biosciences) and Illumina Hiseq 2500 technologies at Biozeron Biotech Co., LTD (Shanghai, China). Genome assembly was *de novo* performed with SOAPdenovo v2.04 [9] and Celera Assembler 8.0 [10]. Putative protein-coding sequences were predicted by Glimmer 3.02 [11]. Gene functional annotation was performed using BlASTP with Nr, String, COG and KEGG databases. rRNA, tRNA were predicted using RNAmmer v1.2 and NCBI Prokaryotic Genome Annotation Pipeline respectively. Protein coding genes were analyzed for COG functional annotation using WebMGA server [12]. CRISPRFinder, freely accessible at http://crispr.i2bc.paris-saclay.fr is used to find clustered regularly interspaced short palindromic repeats (CRISPRs) in *S*. *niveus* SCSIO 3406 genome. Genes involved in secondary metabolic pathways were predicted using antiSMASH 2.0 (http://antismash.secondarymetabolites.org/) [13].

## Results and discussion

**Fig 2:**
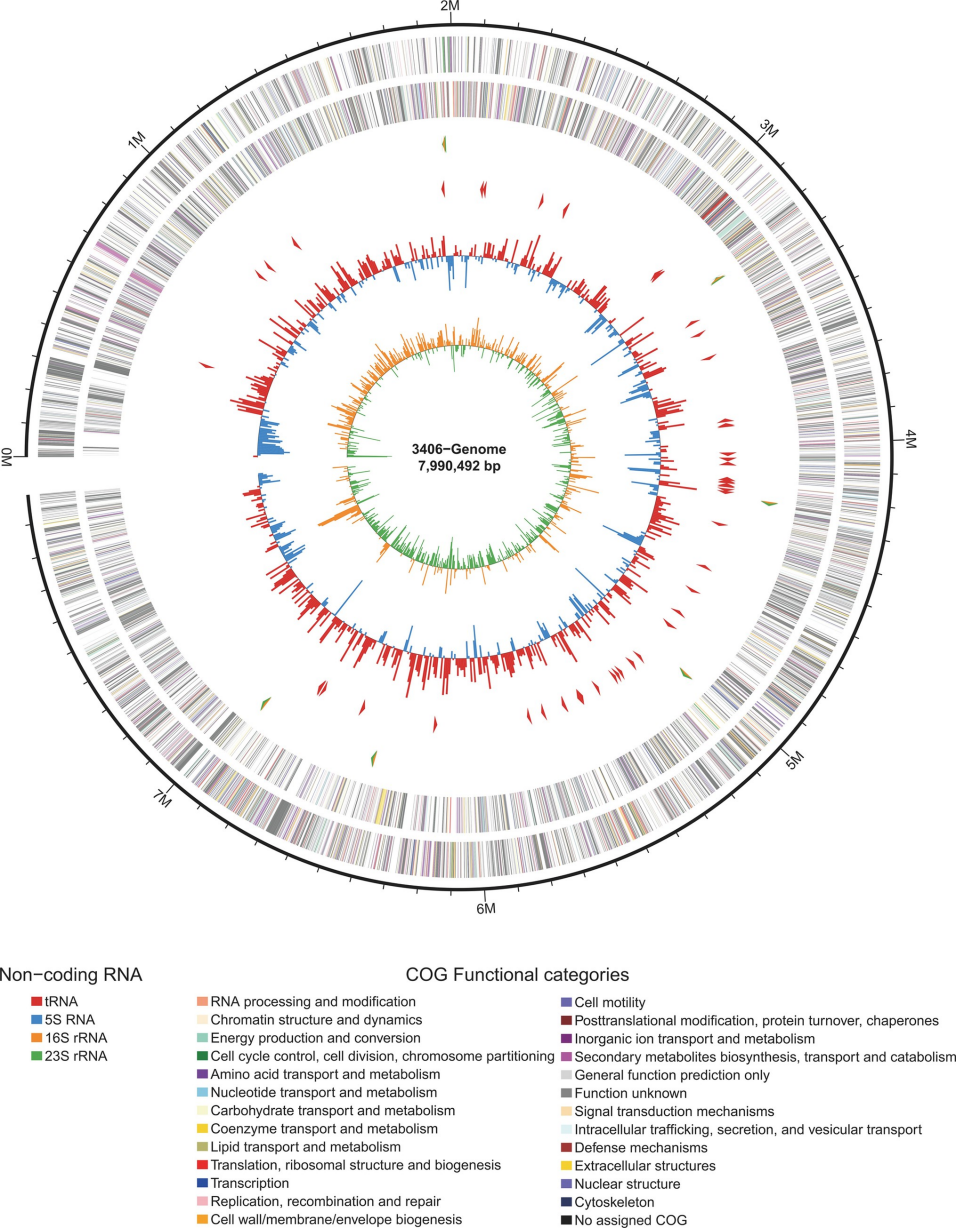
Genome map of *S*. *niveus* SCSIO 3406. The genome map consists of six circles. From outside to inside: (1) the outer two circles show COG functional categories of coding regions in both the forward and reverse strands; (2) the third circle shows the location of rRNA genes; (3) the forth circle shows the location of tRNA genes; (4) the fifth circle shows GC content values; (5) the central circle shows GC-skew values (G−C/G+C) of the third bases of codons measured over the genome.

The complete genome of the strain *S*. *niveus* SCSIO 3406 consisted of a single linear chromosome of 7,990,492 bp with an average GC content of 70.46% without plasmids. Annotation revealed a total of 7,088 protein-coding genes, 6 rRNA operons with the order 5S-16S-23S, and 65 tRNA genes which could transfer all the twenty proteinic amino acids (Table 1, Fig 2). Of the 7,088 protein-coding genes, 3345 (38.3%) were classified into different COG functional categories (Table 2, Fig 2), 4157 shared significant homology with genes from *S*. *niveus* NCIMB 11891, the producer of the gyrase inhibitor novobiocin [14]. Most of the genes in *S*. *niveus* SCSIO 3406 were associated with functions such as “transcription” (K), “amino acid transport and metabolism” (E), “carbohydrate transport and metabolism” (G), “Energy production and conversion” (C), and in particular, “Secondary metabolites biosynthesis, transport and catabolism” (Q) (Table 2).

**Table 1:** Genomic features of *S*.*niveus* SCSIO 3406.

Features	Values
genome topology	linear
genome size (bp)	7,990,492
the sequencing coverage	315.5
average G+C content (%)	70.46
protein-coding genes	7088
rRNA genes	6, 6, 6 (5S, 16S, 23S)
tRNA genes	65

**Table 2:** COG functional categories of the complete genome sequences of *S*. *niveus* SCSIO 3406.

COG code	Description	Number of unigenes
B	Chromatin structure and dynamics	1
J	Translation, ribosomal structure and biogenesis	129
K	Transcription	373
L	Replication, recombination and repair	129
D	Cell cycle control, cell division, chromosome partitioning	17
M	Cell wall/membrane/envelope biogenesis	152
O	Posttranslational modification, protein turnover, chaperones	86
T	Signal transduction mechanisms	193
U	Intracellular trafficking, secretion, and vesicular transport	20
V	Defense mechanisms	86
C	Energy production and conversion	195
E	Amino acid transport and metabolism	349
F	Nucleotide transport and metabolism	56
G	Carbohydrate transport and metabolism	345
H	Coenzyme transport and metabolism	108
I	Lipid transport and metabolism	134
P	Inorganic ion transport and metabolism	186
Q	Secondary metabolites biosynthesis, transport and catabolism	118
R	General function prediction only	489
S	Function unknown	179

Both marfuraquinocins A-D and phenaziterpenes A-B have a moiety of terpene. All terpenoids are synthesized from two precursors: isopentenyl diphosphate (IPP) and dimethylallyl diphosphate (DMAPP). Two distinct biosynthetic pathways produce the essential primary metabolites IPP and DMAPP: the 2-C-methylerythritol 4-phosphate (MEP) pathway and the mevalonate pathway (MVA) [15]. IPP isomerase catalyzes the interconversion of IPP and DMAPP [16]. Based on the Nr annotation, no unigenes were identified as the genes of MVA pathway. However, seven genes scattered around the genome were identified as being involved in the MEP pathway: *orf00197* coding for 1-deoxy-D-xylulose-5-phosphate synthase, *orf01743* coding for 1-deoxy-D-xylulose-5-phosphate reductoisomerase, *orf03854* coding for 2-*C*-methyl-D-erythritol 4-phosphate cytidylyltransferase, *orf04260* coding for 4-diphosphocytidyl-2*C*-methyl-D-erythritol kinase, *orf03855* coding for 2-*C*-methyl-D-erythritol 2,4-cyclodiphosphate synthase, *orf04113* coding for 4-hydroxy-3-methylbut-2-en-1-yl diphosphate synthase, and *orf04123* coding for 4-hydroxy-3-methylbut-2-enyl diphosphate reductase. This implies that the isoprenoid moieties of marfuraquinocins A-D and phenaziterpenes A-B are derived from MEP.

**Fig 3:**
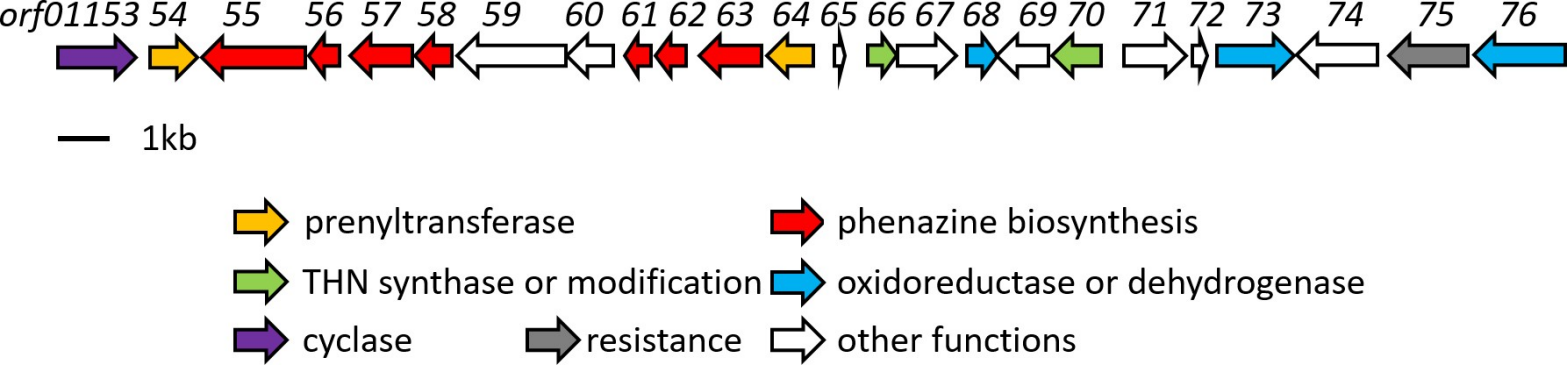
Organization of the gene cluster of compounds 1–6 in *S*. *niveus* SCSIO 3406.

We identified gene *orf01170* coding for type III polyketide synthase and *orf01166* for 1,3,6,8-tetrahydroxynaphthalene (THN) monooxygenase, responsible for the formation and modification of THN successively [17] to generate THN moiety of marfuraquinocins A-D. In many cases, the encoding genes responsible for the antibiotic biosynthesis are clustered in a continuous genomic DNA region, and usually in association with one or more genes that regulate their transcription and with resistance genes [18]. Therefore, the other biosynthetic genes for marfuraquinocins A-D are expected to exist in the vicinity of *orf01170* and *orf01166*. By bioinformatic analysis of the genes located upstream and downstream of *orf01170* and *orf01166*, a ~28.7 kb continuous DNA segment encoding for 24 open reading frames (ORFs) was predicted to contain the putative gene cluster of marfuraquinocins A-D. The function of the individual ORFs was deduced by BLAST analysis. The results are summarized in Table 3 and Fig 3. The identified DNA segment also included a squalene-hopene cyclase gene (*orf01153*), which is predicted to catalyze the cyclization of sesquiterpene to generate one of the parent skeletons of marfuraquinocins A-D. In addition, we found that, as shown in Fig 3 and Table 3, this continuous DNA segment also contained orthologues of most genes required for phenazine biosynthesis in two discontinuous loci (*orf01155-01158* and *orf01161*-*orf01163*). Although the orthologue of *phzF* necessary for phenazine biosynthesis is absent in this DNA segment, two *phzF* orthologues (*orf05123* and *orf05140)* were identified outside of the gene cluster.

**Table 3:** Deduced function of the open reading frames in Fig 3.

ORFs	Amino acids	Proposed function	Sequence similarity (protein accession number, origin,similarity/identity)
*orf01153*	498	squalene-hopene/tetraprenyl-beta- curcumene cyclase	SFG55119.1, *Streptomyces mirabilis*, 53/42
*orf01154*	305	ABBA prenyltransferase	AFS18550.1, *Streptomyces tendae*, 77/60
*orf01155*	657	anthranilate synthase, 2-amino-2- desoxy-isochorismate synthase, PhzE	CDH35391.1, *Streptomyces iakyrus*, 87/83
*orf01156*	207	isochorismatase, also known as 2, 3 dihydro-2, 3 dihydroxybenzoate synthase, PhzD	WP_033308464.1, *Streptomyces iakyrus*, 89/83
*orf01157*	390	3-deoxy-D-arabino-heptulosonic acid 7- phosphate synthase, PhzC	CDH35389.1, *Streptomyces iakyrus*, 88/83
*orf01158*	234	phenazine biosynthesis protein A/B	CDH35388.1, *Streptomyces iakyrus*, 94/88
*orf01159*	704	membrane protein	CDH35387.1,*Streptomyces iakyrus*, 90/83
*orf01160*	294	RNA polymerase sigma factor	SER87811.1, *Streptomyces* sp. Yr375, 86/75
*orf01161*	168	phenazine biosynthesis protein A/B	CDH35386.1, *Streptomyces iakyrus*, 92/86
*orf01162*	216	FMN-dependent oxidase, PhzG	CDH35385.1, *Streptomyces iakyrus*, 89/84
*orf01163*	408	salicylate hydroxylase, PhzS	CDH35383.1, *Streptomyces iakyrus*, 85/79
*orf01164*	301	aromatic prenyltransferase	CDH35382.1, *Streptomyces iakyrus*, 84/70
*orf01165*	68	hypothetical protein	WP_062209276.1, *Streptomyces* sp. NBRC 109706, 78/65
*orf01166*	185	1,3,6,8-tetrahydroxynaphthalene monooxygenase	SDJ65950.1, *Actinopolyspora mzabensis*, 84/74
*orf01167*	380	pyridoxal phosphate-dependent aminotransferase	WP_049565889.1, *Streptomyces* sp. SBT349, 93/87
*orf01168*	203	NAD(P)H:quinone oxidoreductase	WP_079314593.1, *Microbispora* sp. GKU 823, 81/71
*orf01169*	332	SAM-dependent methyltransferase	WP_073501777.1, *Streptomyces paucisporeus*, 85/76
*orf01170*	317	type III polyketide synthase	SHN18252.1, *Streptomyces paucisporeus*, 90/83
*orf01171*	404	putative transcriptional regulator	CDH35377.1, *Streptomyces iakyrus*, 77/71
*orf01172*	100	hypothetical protein	WP_047018058.1, *Streptomyces* sp. CNQ-509, 74/57
*orf01173*	505	FAD/FMN-containing dehydrogenase	SFF48976.1, *Streptomyces alni*, 67/53
*orf01174*	520	long-chain acyl-CoA synthetase	WP_047015640.1, *Streptomyces* sp. CNQ- 509, 82/74
*orf01175*	509	Puromycin resistance protein pur8	AKH84805.1, *Streptomyces* sp. CNQ-509, 80/70
*orf01176*	588	FAD-dependent oxidoreductases	WP_073501787.1, *Streptomyces paucisporeus*, 87/79

Two prenyltransferase genes (*orf01154* and *orf01164*) were identified in this DNA segment. Prenyltransferases are a class of enzymes that transfer allylic prenyl groups to acceptor molecules [19]. Therefore, *orf01154* and *orf01164* were predicted to be responsible for the condensation reaction between the sesquiterpene moiety and the THN moiety to form the backbone of marfuraquinocins A-D and/or the condensation reaction between the monoterpene moiety and the phenazine moiety to form the backbone of phenaziterpenes A-B. Based on above all, we speculate that the continuous DNA segment of ~28.7 kb was involved in the biosynthetic pathway of both marfuraquinocins A-D and phenaziterpenes A-B.

Outside the gene cluster, we also identified gene *orf00932* encoding isopentenyl pyrophosphate isomerase (IPP isomerase). In particular, six geranyl diphosphate (GDP) synthase and farnesyl diphosphate (FDP) synthase genes (*orf00192*, *orf00915*, *orf01258*, *orf02165*, *orf04121* and *orf06133*) are located outside the ~28.7 kb gene cluster. GDP synthase and FDP synthase catalyze the addition of one and two molecules of IPP to DMAPP, yielding GDP and FDP, respectively [20]. Therefore, these six GDP/FDP synthases are proposed to be responsible for the formation of terpene core moieties of both marfuraquinocins A-D and phenaziterpenes A-B. It is obvious that the genes of the biosynthesis of marfuraquinocins A-D and phenaziterpenes A-B are not clustered at a single locus of the genome.

The major interest of *Streptomyces* is its potential to produce diverse secondary metabolites with biological activities. Here, analysis using antiSMASH showed 27 other gene clusters in the genome of *S*. *niveus* SCSIO 3406 (Table 4). Of these gene clusters, some have the really low similarity with the known clusters, revealing the potential of *S*. *niveus* SCSIO 3406 to produce novel natural products. In the putative T1pks-oligosaccharide gene cluster (Table 4, cluster_21), about 32% gene coding products showed similarity with the homologues of the known biosynthetic gene cluster of angucycline antibiotic, grincamycin, in *Streptomyces lusitanus* SCSIO LR32 [21]. We also identify an ectoine gene cluster (Table 4, cluster_23) which consists of hydroxylase (Orf05563), L-ectoine synthase (Orf05564), diaminobutyrate-2-oxoglutarate transaminase (Orf05565) and L-2,4-diaminobutyric acid acetyltransferase (Orf05566) in *S*. *niveus* SCSIO 3406; these gene coding products shows 75% similarity with the homologues of the known ectione biosynthetic cluster (BGC0000853_c1). As one kind of compatible solute, ectoine can be used for protecting enzymes, membranes and whole cells against stresses [22].

**Table 4:** Putative gene cluster coding for secondary metabolites in *S*. *niveus* SCSIO 3406 via antiSMASH.

No.	Type	From (bp)	To (bp)	Most similar known biosynthesis cluster	Similarity
1	NRPS-T1pks	16717	104129	Livipeptin	100
2	Other	184608	228006	_	_
3	NRPS-Linaridin	239107	354223	Laspartomycin	11
4	Terpene-otherks-T1pks	425830	492314	Carotenoid	54
5	T3pks	515177	556304	Herboxidiene	2
6	Terpene-otherks-lantipeptide	629756	732288	Coelichelin	100
7	Terpene	1046670	1073438	Hopene	69
8	terpene-Bacteriocin	1148109	1182287	_	_
9	Terpene	1439858	1461219	_	_
10	Bacteriocin	1563157	1574539	_	_
11	Terpene	1734097	1755776	_	_
12	butyrolactone	1763284	1774216	_	_
13	Siderophore	1883945	1898683	_	_
14	Other	2284811	2326703	Stambomycin	12
15	Siderophore	3740889	3756085	Macrotetrolide	33
16	Terpene	4086055	4109186	_	_
17	Terpene	4654606	4675796	Merochlorin	7
18	Bacteriocin	4774816	4785109	_	_
19	thiopeptide-lantipeptide	4856826	4891992	_	_
20	butyrolactone	5081056	5092780	_	_
21	T1pks-oligosaccharide	5780811	5848626	Grincamycin	32
22	Nrps	6228545	6274913	_	_
23	Ectoine	6339095	6349493	Ectoine	75
24	NRPS-T1pks	6640764	6720100	Streptomycin	19
25	otherks-Nrps	6975232	7045695	Naphthyridinomycin	92
26	T3pks	7312845	7353951	Alkylresorcinol	100
27	Melanin	7672825	7683463	Melanin	40

CRISPR (Clustered regularly interspaced short palindromic repeat) acronym was proposed by Jansen et al. [23]. CRISPR was observed first in 1987 in *Escherichia coli* [24] and were subsequently reported in a wide range of prokaryotic genomes. CRISPR associated proteins (Cas) use the CRISPR spacers to recognize and cut foreign genetic elements [25]. Therefore, the CRISPR/Cas system is a prokaryotic immune system [26]. Here, 22 CRISPRs candidates spreading over *S*. *niveus* SCSIO 3406 genome, including 6 confirmed CRISPRs and 16 questionable CRIPSRs [27], were identified in *S*. *niveus* SCSIO 3406 genome via CRISPRFinder, well above the average level in *Streptomyces* whose genome sequences have been published (Table 5). The CRISPR sequence contains 168 spacer sequences in size from 19 to 99 bp. The number of spacers in each locus varies from 1 to 78. Only two of the spacer sequences (5’-gcgcgacggacgcgccgccggtgagcacgcgcaggg-3’ and 5’-gtcctcggtccgttcgtcctgcgcgatctccag-3’), namely, protospacers match any sequences in the public sequence databases. The rest of the spacers remain the CRISPR “dark matter”. We also identified ten genes (*orf03151*, *orf05316*, *orf06507-orf06514*) coding for CRISPR-associated proteins. CRISPR loci together with *cas* (CRISPR-associated) genes form the powerful immune system for *S*. *niveus* SCSIO 3406.

**Table 5:** Number of CRISPRs in *Streptomyces* genome.

Strains	Number of confirmed CRISPRs	Number of questionable CRISPRs	Total
*Streptomyces albus* J1074	0	0	0
*Streptomyces avermitilis* MA-4680	3	4	7
*Streptomyces bingchenggensis* BCW-1	3	7	10
*Streptomyces cattleya* NRRL 8057	2	8	10
*Streptomyces coelicolor* A3(2)	1	2	3
*Streptomyces collinus* Tu 365	0	4	4
*Streptomyces davawensis* JCM 4913	0	0	0
*Streptomyces flavogriseus* ATCC 33331	4	2	6
*Streptomyces fulvissimus* DSM 40593	0	0	0
*Streptomyces griseus* subsp. griseus NBRC 13350	3	7	10
*Streptomyces hygroscopicus* subsp. jinggangensis 5008	9	4	13
*Streptomyces hygroscopicus* subsp. jinggangensis TL01	0	0	0
*Streptomyces rapamycinicus* NRRL 5491	10	4	14
*Streptomyces scabiei* 87.22	0	2	2
*Streptomyces* sp. PAMC26508	0	0	0
*Streptomyces* sp. SirexAA-E	0	4	4
*Streptomyces venezuelae* ATCC 10712	0	6	6
*Streptomyces violaceusniger* Tu 4113	5	12	17
*S*. *niveus* SCSIO 3406	6	16	22

Interestingly, several antibiotic resistance genes were identified in *S*. *niveus* SCSIO 3406 genome. The gene *orf04161* encodes penicillin amidase catalyzing the hydrolysis of benzylpenicillin [28], that efficiently accounts for the fact *S*. *niveus* SCSIO 3406 can survive on plates which contained 100μg/ml penicillin; the gene *orf00505* encodes erythromycin esterase hydrolyzing the lactone ring of the 14 membered macrolides erythromycin and oleandomycin [29]; the gene *orf02495* encodes virginiamycin B lyase inactivating the type B streptogramin antibiotics by linearizing the lactone ring at the ester linkage [30].

In summary, we have completely sequenced the genome of the deep South China Sea-derived *S*. *niveus* SCSIO 3406. By bioinformatic analysis, we have identified a biosynthetic gene cluster for both the marfuraquinocins A-D and phenaziterpenes A-B. The identified gene cluster provides important genetic basis for better understanding of biosynthesis mechanism of the marfuraquinocins A-D and phenaziterpenes A-B, as well as the construction of improved strain for the industrial production. Notably, 27 other gene clusters were also predicted in the genome of *S*. *niveus* SCSIO 3406. More importantly, some of these clusters have the really low similarity with the known clusters, strongly suggesting the potential of *S*. *niveus* SCSIO 3406 to produce diversity of novel natural products. This sequence information paves the way for the genome mining of *S*. *niveus* SCSIO 3406 for the novel natural product discovery.
